# Development of an ecological momentary assessment scale for appetite

**DOI:** 10.1186/s13030-014-0029-6

**Published:** 2015-01-15

**Authors:** Hiroe Kikuchi, Kazuhiro Yoshiuchi, Shuji Inada, Tetsuya Ando, Yoshiharu Yamamoto

**Affiliations:** Department of Psychosomatic Research, National Institute of Mental Health, National Center of Neurology and Psychiatry, 4-1-1 Ogawa-Higashi, Kodaira, Tokyo 187-8553 Japan; Department of Stress Sciences and Psychosomatic Medicine, Graduate School of Medicine, The University of Tokyo, 7-3-1 Hongo, Bunkyo-ku, Tokyo 113-8655 Japan; Educational Physiology Laboratory, Graduate School of Education, The University of Tokyo, 7-3-1 Hongo, Bunkyo-ku, Tokyo 113-0033 Japan

**Keywords:** Appetite, Ecological momentary assessment, Food diary, Multilevel factor analysis

## Abstract

**Background:**

An understanding of eating behaviors is an important element of health education and treatment in clinical populations. To understand the biopsychosocial profile of eating behaviors in an ecologically valid way, ecological momentary assessment (EMA) is appropriate because its use is able to overcome the recall bias in patient-reported outcomes (PROs). As appetite is a key PRO associated with eating behaviors, this study was done to develop an EMA scale to evaluate the within-individual variation of momentary appetite and uses this scale to discuss the relationships between appetite and various psychological factors.

**Methods:**

Twenty healthy participants (age 23.6 ± 4.2 years old) wore a watch-type computer for a week. Several times a day, including just before and after meals, they recorded their momentary psychological stress, mood states, and ten items related to appetite. In addition, they recorded everything they ate and drank into a personal digital assistant (PDA)-based food diary. Multilevel factor analysis was used to investigate the factor structure of the scale, and the reliability and validity of the scale were also explored.

**Results:**

Multilevel factor analyses found two factors at the within-individual level (hunger/fullness and cravings) and one factor at the between-individual level. Medians for the individually calculated Cronbach’s alphas were 0.89 for hunger/fullness, 0.71 for cravings, and 0.86 for total appetite (the sum of all items). Hunger/fullness, cravings, and total appetite all decreased significantly after meals compared with those before meals, and hunger/fullness, cravings, and total appetite before meals were positively associated with energy intake. There were significant negative associations between both hunger/fullness and total appetite and anxiety and depression as well as between cravings, and depression, anxiety and stress.

**Conclusions:**

The within-individual reliability of the EMA scale to assess momentary appetite was confirmed in most subjects and it was also validated as a useful tool to understand eating behaviors in daily settings. Further refinement of the scale is necessary and further investigations need to be conducted, particularly on clinical populations.

## Background

Understanding eating behaviors is important for the health education of healthy people as well as for treatment in diverse clinical populations, which range from obesity to eating disorders. Although psychosocial factors have been identified as having a significant effect on eating behaviors in previous studies in laboratory analyses or from recalled self-reports [1-3], ecological validity or the validity as to how well the data reflects actual daily life was not necessarily guaranteed. Ecological momentary assessment (EMA) is now used to investigate the psychosocial factors related to eating behaviors to achieve high ecological validity [4,5]. EMA is an assessment method in which participants record their momentary states in daily settings [6], and has been mainly used for patient-reported outcomes (PRO) such as psychosocial factors. Recently, mobile computers have been used as the recording devices because these are able to timestamp the recordings to ensure an accurate time axis by avoiding “faked compliance”.

With regard to nutritional assessment of eating behaviors, the 24-hour recall method has been regarded as the gold standard [7]. For assessment over a prolonged period, paper-and-pencil diaries in which participants record their food intake and calculate nutritional intake have been widely used. Problems such as faked compliance and respondent burden have plagued such studies in the past. Therefore, we developed a personal digital assistant (PDA)-based food recording system with photos in their database and validated its accuracy by comparing it with the 24-hour recall method [7]. This PDA-based system and EMA are considered ecologically valid, viable, assessment tools for the investigation of eating behaviors.

Appetite is another important PRO because it can affect eating behaviors and is thought to be affected by both physiological and psychological factors. Appetite is a subjective sensation and can vary considerably from moment to moment, similar to other PROs such as pain and mood states. Therefore, when investigating detailed changes in appetite, recall bias is often problematic; thus, the use of an EMA is beneficial to gain a more accurate picture. When incorporating appetite assessment into EMA to capture within-individual appetite changes and to investigate the temporal relationships with eating behaviors and psychosocial factors, there are two issues that need to be addressed in terms of the appetite scale. The first is the lack of any rigorous investigation on a suitable appetite scale factor structure. Appetite has been generally assessed using words related to appetite (e.g., hunger, fullness, and desire to eat) as single-item questions with visual analog scales (VAS) [8-12]. Responses were used as variables item-by-item instead of using a composite variable based on a factor structure, as is common with ordinary questionnaires. The second issue is the need to consider psychometric properties when using the EMA platform. In contrast to the usual questionnaires, EMA recordings are repeated by the same individual over a short time and can then be used for within-individual comparisons as well as for between-individual comparisons. Because of this, the psychometric properties when a scale is used in the form of EMA do not necessarily resemble those when it is used in the form of usual questionnaires, and they should be investigated based on the EMA data collected in daily settings [13]. The electronic form of the VAS has been investigated for use in appetite assessment to determine if it is reliable in comparison with the paper and pencil form [14-16]. However, other psychometric properties such as internal consistency and validity have not been investigated in daily settings.

Therefore, this study developed an EMA scale to evaluate the within-individual variation in momentary appetite and discusses the psychometric properties and their relationship with psychological factors based on the EMA data collected in daily settings.

## Methods

### Participants & procedures

In the autumn of 2008, 20 healthy undergraduate and graduate students were recruited for participation in this study. All participants were Japanese and belonged to universities in the Tokyo metropolitan area. The inclusion criterion was age ≥ 20 years, and the exclusion criteria were the presence of any physical or mental disorders, any history of an eating disorder, or being on a diet. Health status and history were based on self-reports. All participants were given a full explanation of the purposes and potential risks of the study by well-trained researchers, and written informed consent was obtained from all participants. The study was approved by the research ethics committee of the Graduate School of Education, the University of Tokyo.

For seven consecutive days the participants wore a watch-type computer (Ruputer ECOLOG; 42 grams, Seiko Instruments Inc., Tokyo, Japan; with a 20 × 30 mm screen, a joystick, and a button) [17,18] and carried a PDA (SL-C3000; 298 grams, 124 × 87 × 25 mm, Sharp Corporation, Osaka, Japan; with 3.7 inches color LCD) [7] as an electronic diary and a food-recording system. Participants were fully instructed on the use of the devices and given manuals.

### Measures

#### Ecological momentary assessment of psychosocial factors and appetite

Momentary psychosocial factors were recorded using EMA. Momentary psychological stress and nine items from the Depression and Anxiety Mood Scale (DAMS) [19] were shown as questions with a VAS displayed on the watch-type computer that ranged from 0 to 100. Depression and anxiety scores were calculated from the nine DAMS items and rescaled to a range of 0 to 100.

Candidate items on the appetite scale were created in the following manner. First, every item used as a single-item question in published papers [8-12] was listed. Then, they were modified in order to avoid redundancy through discussions with the authors. These items were hunger, fullness, prospective food consumption, thirst, nausea, urge to eat, eagerness to eat, desire to eat something sweet, desire to eat something savory, and preoccupation with thoughts of food. Each item was shown as a question on the VAS as described above. The anchor words were “none” and “most intense”.

The EMA recordings comprised signal-contingent recordings and event-contingent recordings. Signal-contingent recordings are recordings that are cued through random alarm alerts at 30-minute intervals around the following times: 5:00, 10:00, 15:00, 20:00, and 24:00. If participants did not enter a recording when the alarm sounded, they were allowed to postpone input for up to 30 minutes. Recordings not made within 30 minutes were cancelled.

Event-contingent recordings are recordings made by the participants themselves when predefined events occur. In this study, they were asked to make recordings 1) when they woke up, 2) when they went to bed, 3) just before eating or drinking, and 4) just after eating and drinking. After they made a recording when they went to bed, the computer suspended the alarms until a recording when they woke up was made.

#### PDA-based food diary

Participants were required to record everything they ate and drank using a PDA-based food diary as soon as possible after having something to eat or drink. To avoid the effect of reflecting on their food intake in terms of their psychological state, participants were asked to make an EMA recording before completing the food diary.

First, the participants input information related to food intake; Starting time, place of food intake, and meal type. Place was chosen from a pull-down menu with the following choices: “Home”, “office/school”, “eating out”, and “others”. Meal types were chosen from a pull-down menu with the following choices: “Breakfast”, “lunch”, “dinner”, and “snack”. Thereafter, the participants selected each menu from the database displayed on the PDA screen. Finally, they adjusted their portion sizes according to the photos in the database and input the ratio of the actual quantity they ate to the quantity shown in the database. In this manner, the PDA-based food diary stored the time of entry, related information, menu, and quantity. The system then automatically calculated the energy, protein, fat, carbohydrate, and sodium intakes.

### Statistical analysis

Because the data in this study had a nested structure due to the between-individual and within-individual levels, multilevel modeling was used for statistical analysis. First, the within- and between-individual variances for each item were estimated for descriptive purposes. Second, the appetite scale factor structure was investigated using exploratory multilevel factor analysis using a weighted least square method with a Geomin rotation. Third, the internal consistency for the scale reliability was examined by calculating the Cronbach’s α individually, rather than using multilevel modeling. Fourth, the changes in the appetite after meals and the association between the energy intake and appetite before food intake were tested to investigate the validity of the scale. Appetite scores just before meals were paired with those just after the same meal if available and the appetite differences were calculated. These differences were treated as random effects, which allowed them to vary between the participants. The variance-covariance matrix (G matrix) was modeled as unstructured. To calculate the association between energy intake and appetite, the energy intake per meal was the dependent variable and each of the three appetite scores (“hunger/fullness”, “cravings”, and “total appetite”) were the independent variable in separate models. To control for the meal type, this was also incorporated into the model. Appetite effects were modeled as random effects and the G matrix was modeled as a variance component structure. Finally, the associations between appetite and mood states before a meal were tested to determine the psychological aspects of appetite. The nominated appetite score (out of the three choices) was used as the dependent variable and a psychological factor (out of depression, anxiety, and psychological stress) was used as the independent variable in separate models. The psychological factor effect was modeled as both fixed and random effects and the goodness of fit was compared between null (without psychological factors), fixed, and random models. The G matrix was modeled as unstructured. Goodness of fit was compared using a −2 log likelihood function and *χ*^2^ test and the significance level was set at 0.05. The statistical analyses were conducted using the SAS Proc Mixed (SAS Enterprise Guide 5.1, SAS Institute Inc., Cary, NC) and Mplus 7.1 (Muthen &Muthen, Los Angeles, CA).

## Results

### Profile of the participants

This study included a total of 20 participants, and their profiles are indicated in Table 1.

**Table 1:** **Profile of the participants**

	**Men (n = 10)**	**Women (n = 10)**
Age (years old)	21.5 ± 0.7	25.7 ± 5.1
Height (cm)	175.9 ± 4.2	162.2 ± 5.1
Weight (kg)	68.8 ± 4.2	55.2 ± 5.43
Body mass index (kg/m^2^)	22.3 ± 1.5	21.0 ± 1.6

### Profile of the recordings

The 20 participants made 1588 EMA recordings over seven days, with 138 recordings being made on awakening, 133 recordings being made at bedtime, 428 recordings being made as a result of the alarm, 455 recordings being made just before meals, and 434 recordings being made just after meals.

In addition, the 20 participants made 509 food diary recordings over seven days, with 106 breakfasts, 126 lunches, 136 dinners, and 141 snacks.

### Within- and between-individual variances for each item

The within- and between-individual variances for the ten items on the appetite scale were estimated using multilevel modeling, the results for which are indicated in Table 2. As expected, most items, except for “nausea”, showed large within-individual variances and small between-individual variances, suggesting that the ratings for these items varied from time to time within a participant, whereas average ratings did not show a large difference between the participants.

**Table 2:** **Mean and within- and between-individual variance of the items related to appetite**

**Item**	**Mean**	**(S.E.)**	**Variance**			
			**Within-individual**	**(S.E.)**	**Between-individual**	**(S.E.)**
Hunger	37.9	(2.3)	666.4	(23.6)	99.3	(33.9)
Fullness	47.7	(1.8)	710.7	(25.1)	54.6	(19.8)
Prospective food consumption	41.1	(2.4)	457.4	(16.2)	105.6	(35.2)
Thirst	47.7	(3.3)	448.8	(15.9)	207.7	(67.3)
Nausea	15.2	(2.6)	151.5	(5.4)	135.8	(43.5)
Urges to eat	33.4	(2.7)	582.5	(20.6)	134.0	(44.8)
Eagerness to eat	27.2	(3.0)	378.6	(13.4)	173.7	(56.5)
Desire to eat something sweet	28.8	(3.3)	402.4	(14.2)	213.5	(69.2)
Desire to eat something savory	21.4	(3.2)	259.0	(9.2)	199.0	(63.9)
Preoccupation with thought of food	20.8	(2.9)	261.4	(9.2)	163.3	(52.7)

### Factor structure (multilevel factor analysis)

Because the primary aim of the scale was to capture a within-individual change in appetite, it first focused on the within-individual factor structure (factor structure at a within-individual level). From the eigenvalues, the scree plot, the goodness of fit and interpretability, a two-factor structure seemed appropriate at the within-individual level. An item with insufficient factor loadings (<0.3) to either factor (“nausea”), items of double loading (both factor loadings ≥ 0.3; “prospective food consumption”), and items with low communality (<0.40; “thirst”, “desire to eat something savory”, and “desire to eat something sweet”) were excluded. The factor structure for the remaining five items is indicated in Table 3. The between-individual factor structure (factor structure at a between-individual level) for the five items was well described as a one-factor structure. Although the factor loading for “fullness” was < 0.3, it was included for consistency with the within-individual level. We named factor I “hunger/fullness” and factor II “cravings” and calculated the 0–100 scores for each factor by averaging the item scores (“fullness” was treated as a reverse item.). In addition, “Total appetite” was calculated as an average of all five items.

**Table 3:** **Multilevel factor structure of the appetite scale**

	**Within-individual level**			**Between-individual level**	
**Item**	**I**_**w**_	**II**_**w**_	**Communality**	**I**_**B**_	**Communality**
Fullness	*−0.91*	0.003	0.82	−0.10	0.01
Hunger	*0.65*	0.29	0.50	0.95	0.90
Eagerness to eat	−0.01	*0.80*	0.63	0.95	0.91
Urge to eat	0.25	*0.72*	0.58	0.90	0.82
Preoccupation with thoughts of food	0.002	*0.66*	0.43	0.90	0.81
Factor contribution	1.30	1.67	2.97	3.44	3.44
Contribution rate (%)	26.0	33.4	59.4	68.9	68.9

RMSEA = 0.048 (90%CI = [0.031, 0.066]), CFI = 0.999.

Correlation between two factors at within-individual level is 0.59.

### Reliability (internal consistency)

The medians and ranges for the individually calculated Cronbach’s α for the “hunger/fullness” score, “cravings” score, and “total appetite” score are indicated in Table 4.

**Table 4:** **Individual Cronbach’s αs for appetite scores**

	**Individual Cronbach’s α**	
	**Median**	**Range**
Hunger/fullness	0.89	0.65 - 0.98
Cravings	0.71	0.29 - 0.98
Total appetite	0.86	0.62 - 0.98

### Validity (appetite change after meals)

Changes in the “hunger/fullness”, “cravings”, and “total appetite” scores were all significantly different from zero (Table 5). Grand means for the appetite scores before and after meals were estimated using multilevel modeling (Table 5). All three appetite scores decreased significantly after food intake.

**Table 5:** **Change in appetite score after meals**

	**Before meals**	**After meals**	**t value**	**p value**
Hunger/fullness (0–100)	62.5	25.7	t(19) = 10.22	<0.0001
Cravings (0–100)	40.0	18.3	t(19) = 6.26	<0.0001
Total appetite (0–100)	49.0	21.3	t(19) = 8.02	< 0.0001

### Validity (association between energy intake and appetite before eating)

There were significant positive associations between energy intake and appetite before meals (Table 6).

**Table 6:** **Energy intake and appetite before meals**

	**Estimate**	**(S.E.)**	**F value**	**p value**
**Energy intake and hunger/fullness**				
Intercept (kcal)	148.6	(55.2)		
Effect of hunger/fullness (kcal/point)	3.2	(1.2)	F(1,425) = 6.58	0.011
Effect of meal types			F(3,53) = 60.99	<0.0001
**Energy intake and cravings**				
Intercept (kcal)	156.8	(48.6)		
Effect of cravings (kcal/point)	4.7	(1.8)	F(1,423) = 6.98	0.009
Effect of meal types			F(3,53) = 68.52	<0.0001
**Energy intake and total appetite**				
Intercept (kcal)	117.8	(56.3)		
Effect of total appetite (kcal/point)	4.7	(1.6)	F(1, 421) = 8.66	0.003
Effect of meal types			F(3,53) = 61.16	<0.0001

### Relationship between appetite and mood states before meals

Depression and anxiety were significantly negatively associated with all three appetite scores, whereas psychological stress was significantly negatively associated only with cravings (Table 7). Models that consider psychological factors as fixed effects fit better than random models, except for the effect of depression on hunger/fullness.

**Table 7:** **Associations between appetite and psychological factors before meals**

	**Estimate**	**(S.E.)**	**F value**	**p value**
**Effect on hunger/fullness**				
Depression (point/point)	−0.39	0.10	F(1,420) = 16.51	<0.0001
Anxiety (point/point)	−0.25	0.05	F(1,420) = 24.78	<0.0001
Psychological stress (point/point)	-	-	-	-
**Effect on cravings**				
Depression (point/point)	−0.17	0.08	F(1,423) = 4.46	0.035
Anxiety (point/point)	−0.10	0.05	F(1,423) = 3.89	0.049
Psychological stress (point/point)	−0.18	0.06	F(1,423) = 8.54	0.004
**Effect on total appetite**				
Depression (point/point)	−0.29	0.06	F(1,419) = 22.52	<0.0001
Anxiety (point/point)	−0.19	0.05	F(1,419) = 16.06	<0.0001
Psychological stress (point/point)	-	-	-	-

Estimates are not shown if a model with a psychological factor as a dependent variable did not have a better fit than the unconditional model.

## Discussion

As indicated in Table 2, the within-individual variance was substantial, in contrast to the small between-individual variance for most items except nausea, which suggests that momentary appetite varies from time to time over the day. Considering low mean for the item “nausea,” it was concluded that this sensation was unusual and may not be a useful measure for the daily variation in appetite.

Two factors were suggested at the within-individual level. Stubbs RJ et al., based on the data of children and adolescents, concluded that six items related to appetite were divided into gut-based sensations and motivation to eat [8]. The results of this study could be interpreted in the same manner, with “hunger” and “fullness” measuring an aspect of gut-based appetite and “eagerness to eat”, “urge to eat”, and “preoccupation with thoughts of food” measuring the motivation to eat. At the between-individual level only one factor was suggested, in contrast with the within-individual level. In other words, these items co-varied differently for the within- and between-individual levels. While people with high hunger/fullness tended to have high cravings, periods during which hunger/fullness was high were not necessarily similar to periods when cravings were high. This type of contrast was revealed using a multilevel factor analysis on the EMA data.

Internal consistency was investigated as the reliability analysis. Considering that the principal use of the scale is to capture the within-individual change of appetite, the Cronbach’s αs were calculated individually. Consequently, the median of Cronbach’s αs were generally good, with the exception of one subject who showed a low α for cravings, which may have been due to a small variance in the craving scores for that participant.

The appetite scale had been previously validated in a laboratory treating eating behaviors. Because we wished to investigate the validity of the appetite scale to capture the change in usual daily settings, we used both the appetite data from the EMA and the food intake data from the PDA-based system. Although daily eating behaviors have been shown to be affected by various factors, which may act as noises in this analysis, the results showed that the appetite scores were reduced after food intake and were significantly positively associated with energy intake. Therefore, the validity for the natural eating behaviors in a daily setting was confirmed.

Psychological factors have been found to affect appetite. The results of this study showed a negative association with appetite, although there was a difference in associations between hunger/fullness and cravings whereby depression and anxiety showed significantly negative associations with all three appetite scores (hunger/fullness, cravings and total appetite), while stress showed a significantly negative association only with cravings. Although the relationships between psychological states and the food intake of healthy participants investigated by either laboratory studies or EMA studies have been inconsistent, suggesting that food intake is decreased with negative mood or stress in some participants while food intake is increased or unchanged in others [4,5,20], there are some reports that participants whose food intake is decreased are dominant [20]. The results of our study were consistent with this, although we investigated psychological states and appetite instead of food intake, which was eating behavior itself. It has also been reported that healthy people with emotional eating (food intake with the intention of coping negative affect) tend to eat more with negative mood and stress [2,21]. In addition, increased negative mood has been reported to precede binge eating in EMA studies of eating disorder patients [22,23]. Therefore, if we focus on emotional eating and binge eating, a different relationship would be observed. In the future, it will be necessary to clarify these associations between the two aspects of appetite and other psychosocial factors, such as environmental factors that may affect appetite, and the associations between the two appetite aspects and biological factors that may affect appetite or may explain the biological background. Furthermore, obtaining a more complete picture of the relationship between appetite, biopsychosocial factors, and eating behaviors in daily settings could positively contribute to the promotion of healthy eating behaviors.

The main limitation of this study was that all participants were healthy young people. Therefore, it will be necessary to investigate if these results can be extrapolated to other populations, such as populations of different ages and occupations, although undergraduates and graduates are thought to be fair because we can speculate that they can eat rather naturally and with less environmental constraint compared to busy working people. Investigation into clinical populations, such as obesity and eating disorders, would also be important. The between-individual factor structure might be different because some eating disorder patients are thought to have persistently intense cravings compared to hunger/fullness. The relationships between psychological states and appetite might also be different, as we discussed before. In addition, although we proposed an appetite scale with two factors of five items, assessment of as few items as possible is preferable because of the need to simultaneously assess many other psychosocial factors. Generally, composite scores derived from several items based on an appropriate factor structure are expected to be more reliable than single-item questions. However, of course it is important to confirm if it is actually more reliable and valid and particularly if its advantages outweigh the burden of responding to several questions. Therefore a careful examination is still necessary to determine if the composite scoring system used in this study is better than a scale with fewer items, especially a scale in which a score for an item is used as the variable itself. As regards the sample size, the average number of recordings per person (within-individual level sample size) was 79.4 for multilevel factor analysis and internal consistency analysis and 25.4 for validity analysis and analysis of the relationship with psychological factors. They are thought to be reasonable numbers to estimate within-individual factor structure and relationships. However, the number of participants (between-individual level sample size) was only 20, which is rather small to obtain a robust factor structure and may compromise the generalizability of the results.

## Conclusions

An EMA scale to assess momentary appetite was proposed. The within-individual reliability was confirmed in most of the participants, and it was also validated against eating behaviors in daily settings. It would be a useful tool when seeking to understand eating behaviors in daily settings from a biopsychosocial viewpoint.

## Abbreviations

EMA: Ecological momentary assessment
DAMS: Depression and anxiety mood scale
PDA: Personal digital assistant
PRO: Patient reported outcome
VAS: Visual analog scale

## References

[1] Fay SH, Finlayson G (2011). Negative affect-induced food intake in non-dieting women is reward driven and associated with restrained-disinhibited eating subtype. Appetite..

[2] Wallis DJ, Hetherington MM (2004). Stress and eating: the effects of ego-threat and cognitive demand on food intake in restrained and emotional eaters. Appetite..

[3] Wallis DJ, Hetherington MM (2009). Emotions and eating. Self-reported and experimentally induced changes in food intake under stress. Appetite..

[4] Grenard JL, Stacy AW, Shiffman S, Baraldi AN, MacKinnon DP, Lockhart G (2013). Sweetened drink and snacking cues in adolescents: a study using ecological momentary assessment. Appetite..

[5] Heron KE, Scott SB, Sliwinski MJ, Smyth JM (2014). Eating behaviors and negative affect in college women’s everyday lives. Int J Eat Disord..

[6] Stone AA, Shiffman S (1994). Ecological momentary assessment (EMA) in behavorial medicine. Ann Behav Med..

[7] Fukuo W, Yoshiuchi K, Ohashi K, Togashi H, Sekine R, Kikuchi H (2009). Development of a hand-held personal digital assistant-based food diary with food photographs for Japanese subjects. J Am Diet Assoc..

[8] Stubbs RJ, Hughes DA, Johnstone AM, Rowley E, Reid C, Elia M (2000). The use of visual analogue scales to assess motivation to eat in human subjects: a review of their reliability and validity with an evaluation of new hand-held computerized systems for temporal tracking of appetite ratings. Br J Nutr..

[9] Adamo KB, Wilson SL, Ferraro ZM, Hadjiyannakis S, Doucet E, Goldfield GS (2014). Appetite Sensations, Appetite Signaling Proteins, and Glucose in Obese Adolescents with Subclinical Binge Eating Disorder. ISRN Obes.

[10] Alkahtani SA, Byrne NM, Hills AP, King NA (2014). Acute interval exercise intensity does not affect appetite and nutrient preferences in overweight and obese males. Asia Pac J Clin Nutr..

[11] Brown RC, McLay-Cooke RT, Richardson SL, Williams SM, Grattan DR, Chisholm AW-AH (2014). Appetite Response among Those Susceptible or Resistant to Obesity. Int J Endocrinol.

[12] Van Strien T, Ouwens MA, Engel C, de Weerth C (2014). Hunger, inhibitory control and distress-induced emotional eating. Appetite..

[13] Kamarck TW, Shiffman SM, Smithline L, Goodie JL, Thompson HS, Ituarte PHG, Krantz DS, Baum A (1998). The Diary of ambulatory behavioral states: a new approach to the assessment of psychosocial influences on ambulatory cardiovascular activity. Technology and methods in behavioral medicine.

[14] Whybrow S, Stephen JR, Stubbs RJ (2005). The evaluation of an electronic visual analogue scale system for appetite and mood. Eur J Clin Nutr..

[15] Rumbold PLS, Dodd-Reynolds CJ, Stevenson E (2013). Agreement between paper and pen visual analogue scales and a wristwatch-based electronic appetite rating system (PRO-Diary), for continuous monitoring of free-living subjective appetite sensations in 7--10 year old childr. Appetite..

[16] Zabel R, Ash S, Bauer J, King N (2009). Assessment of subjective appetite sensations in hemodialysis patients. Agreement and feasibility between traditional paper and pen and a novel electronic appetite rating system. Appetite..

[17] Kikuchi H, Yoshiuchi K, Ohashi K, Yamamoto Y, Akabayashi A (2007). Tension-type headache and physical activity: an actigraphic study. Cephalalgia..

[18] Kikuchi H, Yoshiuchi K, Yamamoto Y, Komaki G, Akabayashi A (2012). Diurnal variation of tension-type headache intensity and exacerbation: an investigation using computerized ecological momentary assessment. Bio Psycho Soc Med..

[19] Fukui I (1997). The Depression and Anxiety Mood Scale (DAMS): scale development and validation. Jpn J Behav Ther..

[20] Macht M (2008). How emotions affect eating: a five-way model. Appetite..

[21] Oliver G, Wardle J, Gibson EL (2000). Stress and food choice. A laboratory study. Psychosom Med.

[22] Munsch S, Meyer AH, Quartier V, Wilhelm FH (2012). Binge eating in binge eating disorder: a breakdown of emotion regulatory process?. Psychiatr Res..

[23] Engelberg MJ, Steiger H, Gauvin L, Wonderlich SA (2007). Binge antecedents in bulimic syndromes: an examination of dissociation and negative affect. Int J Eat Disord..

